# Interventions to improve human papillomavirus vaccination among Chinese female college students: study protocol for a randomized controlled trial

**DOI:** 10.1186/s12889-019-7903-x

**Published:** 2019-11-21

**Authors:** Mingyu Si, Xiaoyou Su, Yu Jiang, Youlin Qiao, Yuanli Liu

**Affiliations:** 1 School of Public Health, Chinese Academy of Medical Sciences &Peking Union Medical College, 5 DongDanSanTiao, Dongcheng District, Beijing, China; 2 Department of Cancer Epidemiology, National Cancer Center/National Clinical Research Center for Cancer/Cancer Hospital, Chinese Academy of Medical Sciences and Peking Union Medical College, Beijing, China

**Keywords:** Human papillomavirus, HPV, Cervical cancer, Vaccination, Intervention, IMB, Chinese, Female

## Abstract

**Background:**

While the HPV vaccines have been approved by the US FDA since 2006, in recent years an increasing number of women are living with cervical cancer globally. Among them, Chinese women have a higher cervical cancer incidence and mortality rate than the global average, with mortality rates being almost twice the global average. However, the current approach of HPV vaccination in China is not satisfactory given the high disease burden of cervical cancer. The current study is a randomized controlled trial designed to identify the barriers and facilitators of HPV vaccination among Chinese female students. This study will also test a health intervention measure via a popular form of new media in order to improve the HPV vaccine uptake under the framework of Information-Motivation-Behavioral skill Model (IMB).

**Methods:**

This investigation is a multicenter, school-based, prospective, randomized, parallel group, double-blind, blank-controlled trial involving a 7-day education intervention with a further 6-month follow-up. We will enroll at least 3360 participants older than 18 years. The enrolled participants will be randomly divided into two groups (1:1 ratio). The intervention group will be offered a 7-day mobile health education, and participants in both groups will fill out 4 questionnaires at the baseline, 1 month, 3 months and 6 months after the intervention. The primary outcome is the difference in HPV vaccination or reservation for the HPV vaccine between the intervention and control groups. Secondary outcomes will include the comparison of (1) knowledge, attitudes, motivation, beliefs and behavioral skill about HPV and cervical cancer prevention, and (2) the willingness to uptake HPV vaccination.

**Discussion:**

This study will examine the theory-based intervention in improving HPV vaccination among Chinese female college students. We will conduct the randomized controlled trial to provide scientific evidence on the potential effect of the IMB theory-based intervention. Findings from this study will contribute to a growing research field which assesses the effectiveness of mobile-based, school-targeted and theoretically guided interventions for promoting HPV vaccination in adolescents.

**Trial registration:**

Chinese Clinical Trial Registry (ChiCTR), ChiCTR1900025476; Registered on 27 August 2019.

## Background

Globally, cervical cancer is the fourth most common malignant tumor in women, with an incidence of about 6.6% and a mortality rate of 7.5% [1]. In 2018, about 570,000 new cases of cervical cancer were diagnosed and 310,000 deaths were reported [1]. Among these, low and middle-income countries account for 86% of new cases and 88% of deaths worldwide [1]. In China, cervical cancer is more prevalent than the global average, with the cancer being the second most common malignant tumor among Chinese women [2]. In 2018, the age-standardized incidence rate of cervical cancer among Chinese females was still as high as 10.7% [3], although China has introduced HPV2 (Cervarix), HPV4 (Gardasil) and HPV9 (Gardasil) to the mainland between 2016 to 2018 to cope with the burden of cervical cancer caused by HPV-16 and HPV-18 [4].

While the safety and efficacy of the HPV vaccine has been widely recognized by authoritative organizations and institutions, HPV vaccination rates are still low in mainland China [5–10]. Data from the National Cancer Center suggests that a lack of knowledge on HPV and cervical cancer may be a barrier to HPV vaccination [10, 11]. Studies have also found that Chinese adolescents have a low awareness of HPV vaccination [12, 13]. Other barriers to cancer prevention for Chinese women include cultural beliefs on sexuality and widespread vaccine hesitation or rejection due to misconceptions about HPV prevention [14, 15]. In addition, HPV vaccination has not been publicly provided in China. Instead, citizens must pay for the HPV vaccine at their own expense.

Despite the importance of the HPV vaccine for cervical cancer prevention, limited information has been available on how to improve HPV vaccination among Chinese people [16, 17]. Before the HPV vaccine got approved in mainland China, an intervention study conducted in the junior middle schools of Chengdu, China, found an increase in student willingness to be vaccinated from 56.5 to 88.4% (*p* < 0.001) after 1-h of health education [16]. Similar to the school-based study, the HPV vaccine acceptability has also been observed to increase among employed women and female undergraduate students after an informative group lecture [17]. However, the results of the above mentioned studies did not include the HPV vaccination rate as one of the assessment indices of the intervention. Thus, there is an urgent need to develop effective intervention strategies to improve HPV vaccination among Chinese women, especially using the HPV vaccine uptake as the primary outcome.

Results from the successful promotion experiences of the HPV vaccine abroad suggest that employing culturally tailored and theory-based education could positively influence participants to receive the HPV vaccine, especially if school-based intervention is targeted at adolescent females [18, 19]. Perhaps this is due to a combination of cultural ideals valuing school as a place for students to gain new knowledge, the emerging sexual needs of students and the higher HPV infection rate among adolescents [20–22]. In addition, given the widespread usage of mobile phones among young adults and the ability of mobile phone technology to overcome location and time restrictions, a mobile-based health intervention could be a low-cost and effective method to reach populations with low HPV vaccination rates [23, 24].

**Fig. 1:**
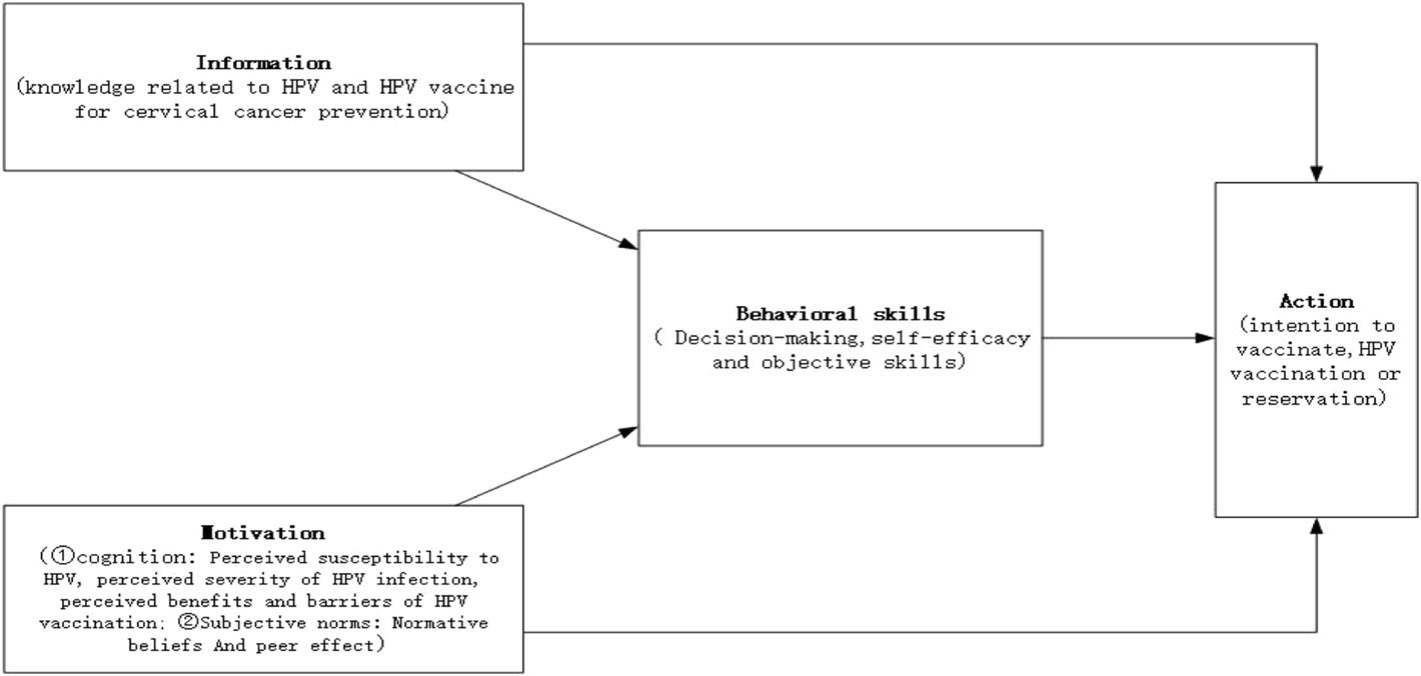
Conceptual Framework of the Study

Given the emerging need for increased HPV vaccination among Chinese women in recent years, the current study will test the effects of a mobile-based intervention on Chinese female college students (see Fig. 1 for conceptual framework), guided by the Information- Motivation- Behavior skill model (IMB) [25]. Specifically designed for the HPV vaccine uptake study, the IMB model constructs used in this study include information relating to HPV and the HPV vaccine for cervical cancer prevention, HPV vaccination motivation, and some useful behavioral skills to ensure HPV vaccination [26].

**Fig. 2:**
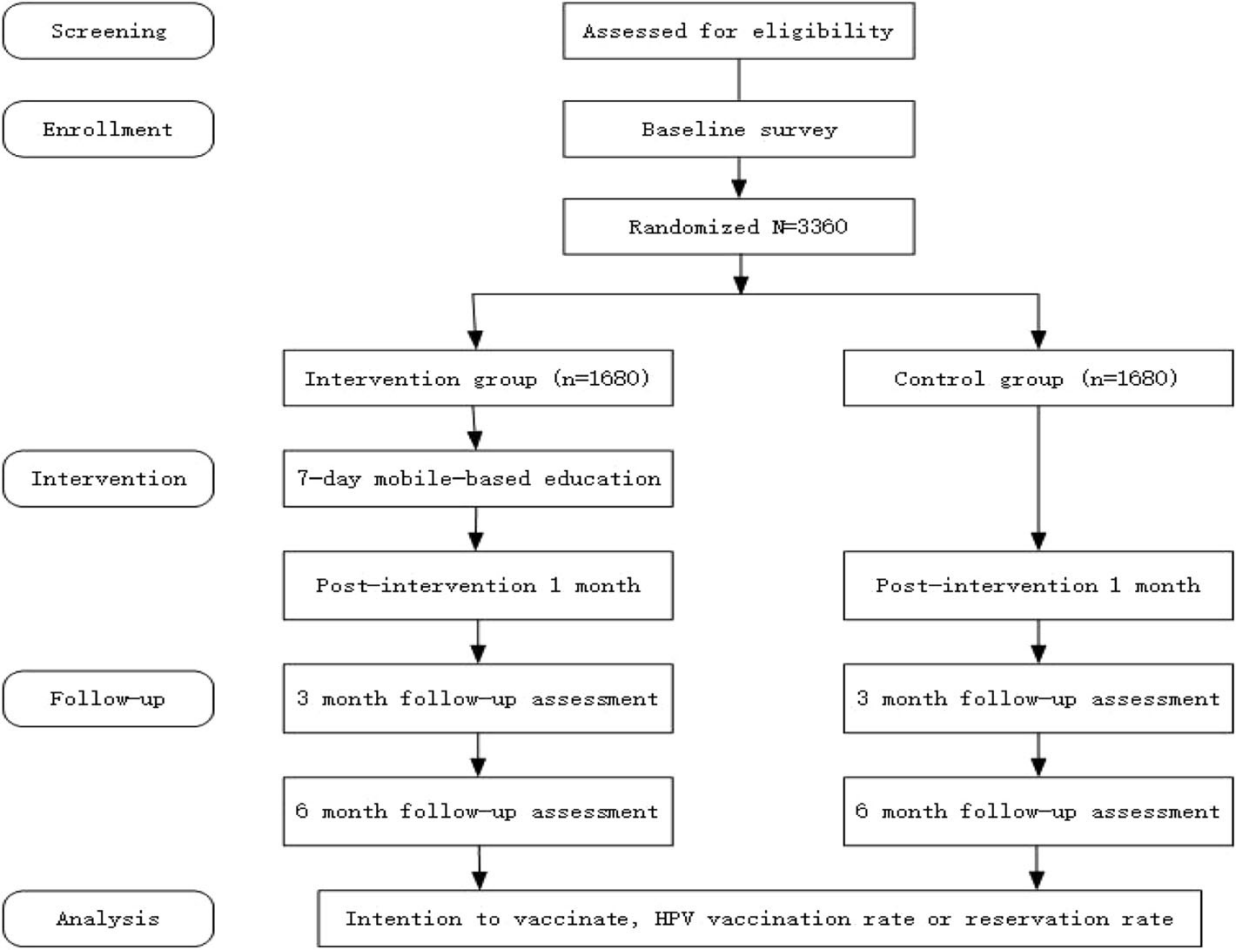
Flow diagram of the study

We hypothesize that a mobile-based approach combining theory-based education and information about HPV, cervical cancer and the HPV vaccine may significantly improve HPV vaccination rates among participants.

Therefore, the primary objective of this study is to investigate the efficacy of a 7-day mobile-based intervention designed to increase HPV vaccination among Chinese female college students. The secondary objectives are to: (1) explore the impact of the intervention on knowledge, attitudes, and beliefs about HPV, cervical cancer and the HPV vaccine, (2) evaluate the short-term effect on uptake intention of the HPV vaccine, (3) determine the factors associated with the willingness of vaccination uptake and (4) identify the factors influencing HPV vaccination.

## Methods/design

### Trial design

This is a multicenter, school-based, prospective, randomized, parallel group, double-blind, blank controlled trial involving a 7-day education intervention with a further 6 months follow-up. A total of 3360 individuals over 18 years old will be randomized to: (1) intervention group: this group will receive a baseline questionnaire followed by a 7-day mobile health education, and 3 questionnaire surveys at 1 month, 3 months, and 6 months after the intervention, or (2) control group: this group will receive 4 questionnaire surveys conducted simultaneously with the intervention group. This study protocol is reported in accordance with the Standard Protocol Items: Recommendations for Interventional Trials statement, and results will be reported in line with the Consolidated Standards of Reporting Trials (CONSORT) statement and the CONSORT statement for non-pharmacological interventions [27–29].

### Study setting

To ensure the representativeness of the research findings, we will recruit participants from seven comprehensive universities located in seven geographical territories of China: Shandong University in East China, Zhongshan University in South China, Zhengzhou University in Central China, Capital Normal University in North China, Sichuan University in Southwest China, Dalian University of Technology in Northeast China, and Xinjiang University in Northwest China. The reasons for selecting these colleges is that they are located in cities with relatively advanced political and economic levels in their respective geographical territories and are all top-ranking comprehensive universities. Thus, the research settings can ensure the balance of background features to a large extent. All researchers and support staff in this project will be trained based on the same training protocols and are required to have an educational background in public health. This study follows the regulations of *Measures for the Ethical Review of Biomedical Research Involving Humans (*Implemented on December 1, 2016) of the National Health Commission of the People’s Republic of China. All participants will sign an informed consent after they agree to attend the study and before the baseline survey. This study is planned to be carried out in November 2019 and last for about 7 months.

### Participants

Suitable participants will be screened at each center for their eligibility to participate in the study:

The inclusion criteria are as follows: (1) females over 18 years of age; (2) first and second-year undergraduate students; (3) no vaccination contraindications; (4) having mobile phone or computer access.

The exclusion criteria are as follows: (1) males; (2) females under 18; (3) non-undergraduate first or second-year students; (4) medical students; (5) previous history of vaccination contraindications; (6) current pregnancy or breast feeding.

### Recruitment and informed consent

All enrolled participants will be informed of the research objectives, the study procedures, their rights and obligations, the expected benefits and the possible risks to the participants before they sign the informed consents (Appendix). Participants in the study will also have the right to freely obtain more information at any time and will be allowed to freely withdraw from the study without restrictions at any research stage.

Once each participant signs the informed consent, the researchers will complete the eligibility checklists and records will be made of any candidate who fails to meet the inclusion criteria. Additionally, in order to facilitate follow-up services, we need participants to leave a mobile phone number or WeChat account. All of the above information is stored on an encrypted laptop and kept by a dedicated person with strict information confidentiality.

### Sampling and randomization

The study process consists of the following four phases:

Phase 1: Confirm the stratified sampling framework by geographical territories of China.

The stratified sampling of the 31 provinces/municipalities/autonomous regions in China is conducted from seven geographical territories, namely East China, South China, Central China, North China, Northwest China, Southwest China and Northeast China.

Phase 2: Convenient sampling to determine the cities and universities to be included within the geographic territories in Phase 1.

We select a representative city in each of the seven geographical territories using the convenient sampling method. A total of 7 cities (Jinan of Shandong Province, Guangzhou of Guangdong Province, Zhengzhou of Henan Province, Beijing, Urumqi of Xinjiang Uygur Autonomous Region, Chengdu of Sichuan Province, Dalian of Liaoning Province) were finally decided to be the study sites. In each representative city, a comprehensive university is selected. A total of 7 universities (Shandong University, Zhongshan University, Zhengzhou University, Capital Normal University, Xinjiang University, Sichuan University, Dalian University of Technology) are finally confirmed as research centers for this study.

Phase 3: Cluster sampling in each of the seven research centers.

After satisfying the inclusion criteria, at least 240 university students will be selected by class from the literature and science majors (excluding medical majors) respectively, and a total of 480 samples will be composed at each of the seven centers. This selection will result in a total of at least 3360 participants across 7 schools.

Phase 4: Randomization and allocation in each center.

After the baseline survey, all identified participants will be randomly assigned to the intervention group or control group in a 1:1 ratio, and each group at each center will have 240 participants. Randomization will be stratified with regards to majors, resulting in two separate categories of literature and science majors. The random sequence will be generated using SAS V.12. (SAS Institute) software. Furthermore, randomization will be conducted by personnel not involved in the trial at each of the seven universities.

After randomization, the researcher who assigns participants to the intervention will provide information about the mobile-based education. This researcher will no longer be blinded from this point.

### Blinding

In this investigation, all researchers involved in outcome assessments will be blinded to the group allocation of participants. During the intervention period, only unblinded researchers will be in contact with participants enrolled in the trial. In addition, all data analysis will be completed by personnel who are blinded to the intervention assignment. Participants will be unblinded after the 6 months and 7 days of assessment.

### Interventions

Theory-based educational measures delivered by a smart application

We developed the contents of the digital multimedia education intervention after reviewing the related literature and conducting several rounds of panel discussions. The panel members included a Cancer Epidemiologist, an Infectious Diseases Epidemiologist, a Senior Manager in a new media company and a researcher from one of the research centers of this project. We also formed a University advisory committee, which consisted of the project leader of each sub-center, authoritative experts in the field of cervical cancer prevention and treatment, and experienced experts in the field of epidemiology and health statistics. Additionally, we conducted a series of focus groups with 50 undergraduate girls and key information interviews with university counselors and health professionals. During such time we modified and refined the intervention content and finally determined that the best format to deliver the 7-day education intervention would be via smart devices such as a computer or mobile phone.

According to the IMB, mobile health intervention popularizes health-related knowledge, develops motivators to raise awareness of healthy behavior, and provides relevant behavioral skills that actually shape specific health behaviors (ie, injection of the HPV vaccine) [26] (see Fig. 1). The educational content of the mobile health intervention will cover the following topics: (1) basic health knowledge including information on the prevention of infectious diseases, vaccination, and sexual health, (2) information on HPV infection, cervix and cervical cancer, and the incidence and mortality rates of cervical cancer among Chinese women compared to the world average level, (3) introduction of the HPV vaccine as a preventive measure for cervical cancer, (4) case study showing the prognosis of Chinese women with advanced cervical cancer, (5) a story telling the experience of a female college student from being unaware of HPV to making a decision to vaccinate against HPV, (6) information on how to improve self-efficacy and self-determination in order to improve healthy behavior, and (7) behavioral techniques for HPV vaccination, including information on the availability and cost of the HPV vaccine at local clinics. The mobile health intervention will be delivered to each participant over 10 min each day for a 7-day period at each participant’s preferred time.

The mobile health intervention contains a high level of interactivity. Participants will be allowed to engage in mutual conversation amongst themselves on different topics of intervention. Based on the mobile application, the intervention group and the control group at each center will establish a chat group respectively, and the administrator will automatically upload 10 min of educational content in the intervention chat group only. After completing the learning session, participants can perform punching and talk about the educational content.

Control group(no intervention/ black control).

### Compliance

Attendance records will be used to assess the participants’ compliance with the intervention. This will occur the following day after each new educational content is uploaded. Furthermore, the number of participants in each questionnaire will be used to assess the participants’ compliance with the entire trial process.

### Procedure

To satisfy the study assessments, participants will complete a total of four electronic questionnaires (see Fig. 2). Electronic surveys will be carried out at approximately the same time (within 1 h) among the participants.

#### Screening

Potential participants will be identified for inclusion/exclusion criteria and those who are eligible will be provided with a written informed consent form. Individuals who agree to attend the study will be briefed on the function of the mobile app and core study measures.

#### Baseline

Prior to the intervention, the baseline survey will be conducted to assess socio-demographic and HPV vaccination status, sexual history, and knowledge, attitudes and beliefs about HPV and HPV vaccination.

#### Intervention procedure

Upon completion of the baseline assessment, two research staff will anonymize and number the students. The staff will then randomize participants to either the intervention group or control group. The intervention and control group will have at least 240 participants respectively at each of the seven centers. Timing randomization in this way ensures we can balance intervention and control groups on baseline characteristics. One unblinded research staff will remain in contact with participants via the mobile app. The intervention group will receive digital education materials every day during the 7-day intervention period, while the control group will not have any form of intervention. Possible intervention non-attendance and trial non-compliance will be collected and followed up via telephone by the unblinded researcher.

#### 1-month follow-up

The participants in both the intervention and control groups will be followed up via the mobile app at 1 month after the intervention, and a questionnaire will be conducted simultaneously. Their HPV vaccination status and HPV related knowledge will be assessed. Likewise, their personal motivation, intention and relevant behavioral skills regarding the injection of the HPV vaccine will also be examined. In addition, the intervention group will assess their satisfaction with the intervention.

#### 3-month follow-up

All measures from the 1-month questionnaire will be repeated in both groups, except for the satisfaction assessment of the intervention.

6-month follow-up: All measures from the 3-month questionnaire will be repeated in both groups, along with a reasons for vaccination refusal inquiry, if required.

### Measures

We will collect information from participants using a structured electronic questionnaire administered at four time points: at the study enrollment (baseline), 1 month after completing the digital education intervention (post-test), 3 months after the intervention and 6 months after the intervention (follow-up).

#### Outcome measures

Our primary outcomes of interest will be the difference between the intervention and the control group, which include: (1) knowledge, attitudes and beliefs about HPV, cervical cancer and the HPV vaccine; (2) intent to uptake the HPV vaccine; and (3) the reception of or an appointment for the first dose of the HPV vaccine.

#### Baseline measures

We will collect participants’ HPV vaccination status, socio-demographic information (date of birth, nationality, religion, habitual residence, economic status, parental employment and marital status, and family or friends’ cancer history), health and sexual history. We will also record information about knowledge, cognition and beliefs related to HPV and HPV vaccination, motivations and intentions to uptake the HPV vaccine, decision-making, self-efficacy and objective skills regarding HPV vaccination. For background information, we will refer to the relevant part of several related studies conducted in China previously. The 11-item scale from Kahn JA and the HAVIQ (HPV Adolescent Vaccine Intervention Questionnaire) from Forster AS will be partly used to measure knowledge about HPV and HPV vaccination [30, 31]. For measuring cognition of HPV infection and prevention, we will use scales from Kim HW, Gerend MA and Guvenc G [32–34]. This will assess the perceived severity of HPV infection and the perceived benefits and barriers of HPV vaccination. Two scales from Ralph will be used to measure the perceived susceptibility of HPV infection [35]. The 5-item scale from Gerend MA will be adopted to test the influence of subjective norms [33]. For measuring decision-making for health protection, we will use the 3-item scale of Forster AS [31]. Self-efficacy for completing the 3-dose HPV vaccination will be tested using the 4-item scale of Gerend MA [33]. The self-administered 4-item scale developed by Fisher will also be used to measure the objective skills needed to initiate and complete HPV vaccination [26]. Finally, intent to receive the HPV vaccine will be measured according to the answer to the question: “Willingness to take the HPV vaccine in the next 6 months”.

#### Post-test measures

At the 1-month follow-up, we will administer a post-test survey of the same information collected at the baseline, except for background information. Furthermore, we will measure participant satisfaction with the mobile app-based education to provide information for improving similar studies in the future.

#### Follow-up measures

At the 3-month follow-up, excluding the satisfaction assessment of the intervention, we will measure the same items collected during the 1-month follow-up test. For participants not receiving or ordering a dose of the HPV vaccine during the trial period, we will ask for the reasons for vaccine hesitation or refusal at the 6-month follow-up survey.

### Sample size calculation

To date, no studies have investigated the effects of a mobile app-based intervention with Chinese female college students to improve the HPV vaccine uptake. For this reason, the anticipated effect size is largely based on findings derived from similar research literature in other countries. This study will apply a two-tail test with α = 0.05 and β = 0.20. Statistical power was originally calculated based on an absolute difference of 10% (20% of girls in the intervention group relative to 10% of girls in the control condition would initiate vaccination). The number of cases needed in each group of each sub-center is 119 as calculated by Statistical Analysis System (SAS) V.12. Allowing for a 20% rate of loss to follow-up, 240 cases are required in each group, and a total of 480 cases are required for each trial center. Consequently, this study requires a total of at least 3360 participants in 7 schools.

### Statistical analysis

Since the study collects data through electronic questionnaires, there will be no formal data monitoring committee. Participants fill out the questionnaire and automatically upload it to a read-only database to ensure the authenticity of the data; however, all data will be reviewed by the research team at regular intervals throughout the study.

We will conduct all analysis using IBM SPSS Statistics 22.0, Version 22.0. Data will be checked for internal consistency and logic prior to further analysis. An intention-to-treat analysis will be performed. Descriptive statistics of all variables will be calculated, including means and standard deviations or frequencies as appropriate. We will assess the potential bias in enrollment and follow-up by comparing characteristics using a Wilcoxon Rank Sum, chi-squared, or Fisher Exact test as appropriate.

Proportions of qualitative variables will be compared between the intervention and control groups using chi-squared tests at baseline and repeated at 1 month, 3 months and 6 months after intervention. For each of these measures, the change in proportion from the baseline to these 3 point-ends will be tested within intervention groups using McNemar’s tests. Unadjusted odds of vaccine uptake and intent to vaccinate for the intervention participants versus the control participants will be calculated using a logistic regression model. Bivariable and multivariable logistic regression models will be used to determine the independent effect of the intervention. Results of logistic regression models will be expressed as odds ratios (ORs) and 95% confidence intervals (95% CIs). Measures of acceptability and satisfaction for intervention participants will be summarized using counts and sample proportions. All final statistical tests are two sided with α = 0.05.

## Discussion

Given that cervical cancer is one of the major causes of death among women worldwide, interventions to prevent high-risk types of HPV (HPV16 and HPV18) infection are urgently warranted. This study will be the first to evaluate whether a mobile-based approach combining theory-based education can improve HPV vaccination among females from mainland China.

Human behavior transformation is a complex process influenced by multiple factors. Therefore, theory-based interventions targeting multiple behavioral factors are needed to address the wide range of mechanisms relevant to the process of understanding HPV to receiving the 3 doses of the HPV vaccine. Promising results have been obtained from the school-based interventions for preventing cervical cancer in adolescents. This used the Health Belief Model (HBM) as a theoretical framework to examine the impact of intervention on HPV vaccination in 741 adolescents aged 16 years [18, 36]. The study design contained four elements: school-based setting, student-target participants, the Health Belief Model (HBM) and a 30 min face-to-face structured review delivered by school nurses. Results of this study demonstrated a significant improvement in the HPV vaccination rate from 52.5% before to 59% after the intervention, whereas no change was seen in the control group (60.9%). These findings are important as they suggest that making well-designed interventions can promote healthy behavior, such as receiving the HPV vaccine for cervical cancer prevention.

Alternatively, the results of DiClemente’s study, which made the vaccination free-of-charge for the intervention group so as to eliminate certain accessibility barriers in a clinical setting, did not show a statistically significant result [35]. Their findings suggest that objective factors, such as vaccine cost and accessibility, may not significantly contribute to poor vaccine uptake. In contrast, a low perceived susceptibility to the HPV infection may help explain the low vaccination rates observed in this study. Furthermore, both the limited sample size and inappropriate recruitment period that conflicted with adolescent’s school hours (9 am-5 pm) had a negative impact on HPV vaccination uptake. Finally, the 12-min media program was likely too short to affect the participants’ desire to receive the HPV vaccine. Thus, similar to the DiClemente’s trial, the current study will be conducted based on the IMB theory. At the same time, however, we will avoid the weaknesses of the above research, as the methodology section demonstrates.

In the current study, we will choose the female college students as the study participants because of their increasing sexual demand and the spread of HPV infection among them [20–22]. School-based interventions are employed as they have been proved suitable and effective in promoting the HPV vaccine in previous studies [16, 18]. In addition, the IMB model is used due to previously beneficial predictions and health behavior promotions across many health areas [35–37]. The Fisher’s recommendations have also been employed due to their usefulness in designing the IMB theory-based interventions to improve HPV vaccination rates in the future. Furthermore, the 7-day education via the mobile app is in line with the preferences of young people [23, 24]. Finally, taking the regional differences into account, our trial will set up seven sub-centers in accordance with the seven geographical divisions in China.

However, this study has potential limitations: First, there is no exact baseline data on vaccination rates to calculate an appropriate sample size, since the HPV vaccine has just been approved in China for just 3 years. Secondly, the short follow-up period of only 6 months and the unverified 7-day education method may also lead to measurement bias. Furthermore, the IMB theory-based questionnaire has not been fully validated among Chinese female college students.

To summarize, this is the first RCT to investigate theory-based interventions to improve human papillomavirus vaccination uptake among Chinese female college students. This multicenter RCT study will contribute to a growing research field which assesses the effectiveness of mobile-based, school-targeted and theoretically guided interventions for promoting HPV vaccination in adolescents.

## Data Availability

Not applicable.

## Appendix

### Model consent form

The school of public health of Peking Union Medical College is carrying out an intervention study on HPV vaccination for Chinese female college students. This study is supported by the Innovative Engineering Program of Chinese Academy of Medical Sciences, and your participation can provide the data needed for the study. To help you understand and make a decision on whether to participate in this study, we will have a specific staff to give you a detailed introduction through this informed consent. If you have any questions, you can raise them at any time.

Human papilloma virus (HPV) is a kind of spherical DNA virus, which can cause the proliferation of squamous epithelium of human skin and mucosa, and lead to genital warts (condyloma acuminatum), cervical cancer, anal cancer, etc. Since July 2016, China has successively introduced three kinds of HPV vaccines to prevent cervical cancer, precancerous lesions and genital warts caused by HPV infection. It has been confirmed that HPV vaccine can effectively prevent the persistent infection of HPV in the world, but it is still recommended that women have cervical examination regularly.

**1. Research purpose:** To understand the current situation of HPV vaccination of female college students in China, to explore the customized electronic education measures based on the information-motivation-behavior skill theory (IMB), and the intervention effect on the HPV vaccination rate of female college students in China, and finally to improve the HPV vaccination rate and reduce the incidence of cervical cancer.

**2. Research methods:** In this study, a certain number of female college students were recruited. After senior staff asked brief screening questions, those who met the selection criteria were recruited. We will collect the changes of awareness, cognition and behavior of subjects on HPV, HPV vaccine and cervical cancer related knowledge through an mobile education intervention and regular e-questionnaire survey. You may be required to download and install the "DingTalk" mobile app, provide your Wechat account and mobile number, so that we can provide e-education and follow-up consultation services.

**3. Rights and Obligations:** Your participation in this study is entirely voluntary. You can choose not to participate in this study, or you can withdraw at any time without any reason, and your rights will not be affected. If you agree to participate in this study, we will conduct e-education and questionnaire survey to understand your situation about HPV related knowledge, cognition and HPV vaccination.

**4. Expected risks and Countermeasures:**

You have the right to refuse to answer some sensitive questions in the questionnaire, or to withdraw from the study at any time.

To provide follow-up services, your mobile number or Wechat account are required. All the above information is stored in an encrypted laptop computer and kept by a specific staff to ensure the confidentiality.

**5. Expected benefits:**

Through this study, the participants can better understand the knowledge about human papillomavirus (HPV), HPV vaccine and cervical cancer prevention. Meanwhile, they will get motivation and the related behavior skills to uptake HPV vaccines. The experience and achievements of the study may provide ideas in promotion of HPV vaccine among female college students, and it will contribute to cervical cancer prevention actions in China.

**6. Expenses:** No chanrge.

**7. Confidentiality agreement:** All your personal privacy information are for research reference only. And the personal data are encoded when applying the information or results.

**8. Contact information:** Please contact us if you have any questions about this research, contact information: .

**9. Signature of informed consent:** I have correctly understood the study, I am voluntary for the research and sign the informed consent before.

## Abbreviations

CIs: Confidence Intervals
FDA: Food and Drug Administration
HPV: Human Papillomavirus
IMB: Information-Motivation-Behavioral skill
OR: Odds ratio
P: Probability
RCT: Randomized Controlled Trial
US: the United States
